# Reduced Leaflet Stress in the Stentless Quadrileaflet Mitral Valve: A Finite Element Model

**DOI:** 10.1371/journal.pone.0067683

**Published:** 2013-07-02

**Authors:** Jian-Gang Wang, Xing-Cheng Kuai, Bi-Qiao Ren, Guang-Fu Gong, Xin-Min Zhou

**Affiliations:** 1 Department of Cardiac Surgery, Beijing Anzhen Hospital, Capital Medical University, Beijing, China; 2 Department of Engineering Mechanics, Hunan University, Changsha, Hunan province, China; 3 Department of Cardiothoracic Surgery, the Second Xiangya Hospital of Central South University, Changsha, Hunan province, China; National Institutes of Health, United States of America

## Abstract

**Background:**

Failure of bioprosthetics is usually caused by calcification of the leaflets as a consequence of high tensile stresses. The stentless valve resembles native mitral valve anatomy, has a flexible leaflet attachment and a suspension at the papillary muscles, and preserves annuloventricular continuity. In this study, the effects of the stentless valve design on leaflet stress were investigated with a finite element model.

**Methods:**

Finite element models of the stentless quadrileaflet mitral valve were created in the close and open configurations. The geometry of the stented trileaflet mitral valve was also analyzed for comparative purposes. Under the designated pressures, the regional stresses were evaluated, and the distributions of stresses were assessed.

**Results:**

Regardless of whether the valve is in the open or close configuration, the maximum first principal stress was significantly lower in the stentless valve than in the stented valve. For the stentless valves, limited stress concentration was discretely distributed in the papillary flaps under both close and open conditions. In contrast, in the stented valve, increased stress concentration was evident at the central belly under the open condition and at the commissural attachment under close condition. In either configuration, the maximum second principal stress was markedly lower in the stentless valve than in the stented valve.

**Conclusions:**

The stentless valve was associated with a significant reduction in leaflet stress and a more homogeneous stress distribution compared to the stented valve. These findings are consistent with recent reports of the clinical effectiveness of the stentless quadrileaflet mitral valve.

## Introduction

Prosthetic cardiac valves are increasingly utilized to treat hemodynamically significant mitral valve disease. Various valve substitutes are commercially available, and are broadly grouped as biological or mechanical prostheses. Mechanical valve prostheses are very durable but carry a higher risk of thromboembolic complications and therefore necessitate life-long anticoagulation. [1] The high risk of life-threatening hemorrhages associated with anticoagulation therapy, especially in elderly patients, is well known. [2] Biological valve prostheses carry a major advantage in that patients are not committed to life-long anticoagulation, but they also have more limited durability than mechanical valves, and so may not be ideal for younger patients. The decreased durability is related in part to the use of a stent, which increases the stress placed on flexion points of the valve leaflet. Furthermore, the frequent bending and shear stress in the leaflets during the opening and closing of the valve has been proposed as a major factor contributing to the calcification process and resultant valve failure [3]–[6].

Therefore, a new valve design offering reduced leaflet stress is likely to prove an important advance in cardiac prostheses. Intuitively, replacement with a bioprosthesis that resembles native mitral valve anatomy might improve valve performance and enhance long-term durability. Recently, a novel stentless bovine pericardial quadrileaflet mitral valve (QMV) has been made available for clinical trials. [7] The pericardium used in the manufacture of this valve undergoes a special anticalcification treatment with polyol, [8] presumably providing a reduced risk of valve failure. The stentless valve is constructed in a manner more similar to the natural mitral valve, consisting of a large anterior, and two commissural and one posterior cusp treated with polyol. The chordal support is concentrated in two flaps for suture to the papillary muscles, so that continuity between the mitral annulus and left ventricle is maintained, leading to a better hemodynamic performance. Furthermore, the absence of rigid stents may allow systolic forces to be absorbed more uniformly within the subvalvular apparatus, which is expected to reduce leaflet stress and improve valve durability.

Failure of bioprosthetic valves is usually caused by leaflet stress and resultant calcification. It is postulated that stentless valves are associated with a reduced leaflet stress, as compared with the traditional stented trileaflet mitral valve (TMV). A three-dimensional finite element model is an effective tool to calculate leaflet stresses in frame-mounted valves [9]–[11]. Therefore, the present study was performed to compare the leaflet stress of the novel stentless valve with a traditional stented valve using a finite element model.

## Materials and Methods

### Production of Valve Prostheses

**Figure 1:**
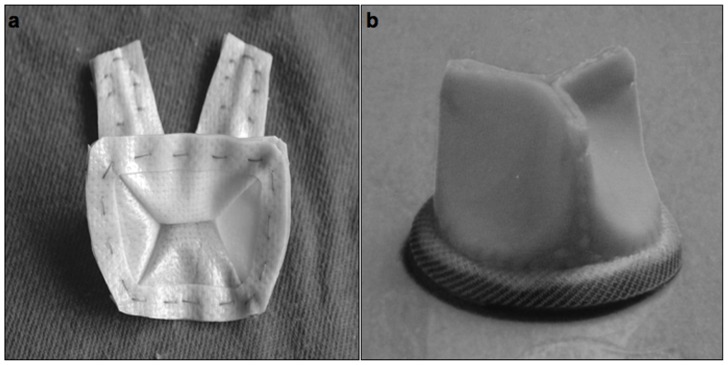
The pictures of the stentless QMV and the stented TMV. The stentless QMV (a) consists of a large anterior and two commissural and one posterior cusp, with two papillary flaps supporting the anterior and posterior leaflet. A stented TMV (b) has a relatively rigid stent in a generally cylindrical configuration with three adjacent semi-lunar leaﬂets.

The stentless QMV (Figure 1a) is made of bovine pericardium with standard glutaraldehyde tanning and additional anticalcification treatment with polyol. The valve consists of a large anterior and posterior leaflet and two small commissural cusps. The leaflet has straight hinge lines to avoid the excessive flexion stress of traditional stented prostheses. The three-layer sewing ring serves as a flexible annuloplasty device. Chordal support consists of two joint papillary flaps that support the anterior and the posterior leaflet.

A stented TMV (Figure 1b) has a relatively rigid stent in a generally cylindrical configuration, with cusps and commissures that are permitted to move radically. The valve consists of three adjacent semi-lunar leaﬂets with uniform thickness, shape, and symmetry. The leaflets are fixed to the stent along a circular curvature.

### Model Geometry and Element Development

Finite element analysis is a computational technique in which an object with a complicated structure is divided into smaller sections (elements) that are interconnected by common points (nodes). This discretization enables the use of algebraic equations to describe the structural state at each node. The solution of the system of equations yields values describing stress and strain at any point in the entire object.

**Figure 2:**
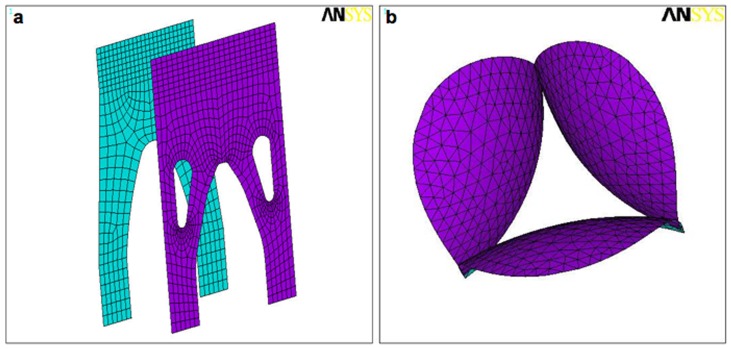
The finite elements mesh divisions of the stentless QMV and the stented TMV. The stentless valve leaflet (a) geometry contains 630 elements, and the stented valve (b) geometry contains 531 elements.

Our finite model was developed using ANSYS software (version 5.3; ANSYS Inc., Canonsburg, PA, USA) run on an Alphastation 400 4/233 (Digital Equipment Corp., Maynard, MA, USA). Cubic splines were fitted to x-y-z coordinates from the stentless and stented valve leaflet geometry, and then meshed to create model nodes. Eight-noded elastic shell elements were utilized in order to model these specific geometries, since elastic shell elements are known to be well suited for reproducing curved geometries. [12] These elements are capable of incorporating both bending and membrane contributions in their prediction of principal stress. The finite element mesh divisions for the stentless and the stented valves are shown in Figure 2a and 2b, respectively. The stentless valve leaflet geometry contains 630 elements; the stented valve geometry contains 531 elements. The mesh density in the vicinity of the commissures was carefully controlled to be consistent for all analyses performed, as it is known that finite element solutions can be highly dependent upon local mesh density.

### Thickness and Material Properties

The thickness of unpressured valve leaflets was determined from previously published data. [13], [14] Based on these reports, the leaflets were set at a uniform thickness of 0.4 mm. The material properties of the leaflet tissues were evaluated according to raw bovine pericardium. Elastic moduli values of 8.75 were determined from linear fits to the physiologic range of the tissue’s stress-strain curves, [15] in directions either parallel or perpendicular to collagen fiber alignment. A Poisson’s ratio of 0.45 was used to represent the nearly incompressible behavior of the valvular tissue. Finally, to represent the pliability of the mitral valve leaflets, the shell elements in the valve were constructed to have a bending stiffness that was reduced by 98.5% as compared with a traditional shell element. A traditional shell element was based on 5-parameter representation of stress in natural coordinates. The stress and strain representations were assumed in terms of skew coordinates.

### Boundary Conditions

To simulate the normal anatomical status of the heart valve prostheses, geometric boundary constraints were assigned. First, three-dimensional contact elements were incorporated into the coapting leaflet surface, to allow for the free sliding of leaflet surfaces and to prevent the model leaflets from passing through each other or through the base plane. Second, the entire structure in the stentless valve was constrained by assigning zero displacement out of the plane on various points of valve rings and the lower edge of tendinous cord, and by allowing its free rotation in any direction. During valve closure process, all parts of the anterior and posterior leaflets were contact elements except valve ring, and the displacement direction was opposite. When valve was at open position, there was no contact element. Third, various points attached to the frame of rigid stent was restricted by arranging in-plane zero displacement and by permitting free circular rotation. At valve open state, contact simulation was not required; while at close state, contact behavior was approximately simulated by setting normal direction displacement of valve to zero in free adjacent area.

### Pressure Loading Pattern

The pressure pattern was designed to model the early diastolic loading of the left ventricle, when peak pressure across the valve is generated. Simulated physiologic pressures were applied to the valve and to the annulus in two phases. In the first phase, the mitral valve was pressurized by a linearly increasing load from the zero-pressure state to left ventricular end-diastolic pressure level. The mitral valve was maintained in the open condition during the left ventricle diastolic period during the entire cardiac cycle. The cardiac output in normal adults was calculated to be approximately 5 L/min. The mean transvalvular pressure gradient in the stentless QMV and the stented TMV were 6.15±0.11 and 8.13±0.03 mmHg, respectively, as determined by fluid dynamic testing. [16] The pressure loading was progressively imposed on the mitral valve from the atrial to ventricular surface under the open condition. In the second phase, the maximum physiologic pressure loads were applied to the valve and the annulus. These physiologic pressures were calculated as the pressure differences between left atrium and left ventricle. Loading was completed at the early stage of left ventricular isovolumic systole, when peak pressure gradient across the mitral valve reached approximately 120 mmHg. The mitral valve was modeled in the closed configuration during left ventricular systole. Pressure loading was rapidly imposed on the mitral valve from the ventricular to atrial surface under the closed configuration.

### Output Analysis

The principal tensile stresses in the valve and papillary flaps were examined at the beginning of physiological loading, when maximum transvalvular pressure occurred in the closed condition. Moreover, the stress was recorded under the open condition of the mitral valve, when the mean transvalvular pressure gradient was applied. Stress (ó) was defined as the force applied to the tissue divided by cross-sectional area, i.e. ó =  F/A. Color contour plots, numerical tabulations, and graphs were employed to aid in the evaluation of data output. Regional magnitudes were calculated by grouping sets of elements to define specific model components, and then analyzing those components to determine the average, maximum and minimum values. In addition, the peak average was calculated, which was defined as the average value of a group containing the top 5% of the elements with the highest values.

### Statistical Analysis

These maximum first and second principle stresses were expressed as mean ± standard deviation. The maximum stresses were obtained with some statistical interval and the range of results reflected the fluctuant alteration on the basis of fixed input parameters. The data were compared using ANOVA to determine significant differences. *P*<0.05 was considered to be statistically significant.

## Results

### Valvular Deformed Configuration

**Figure 3:**
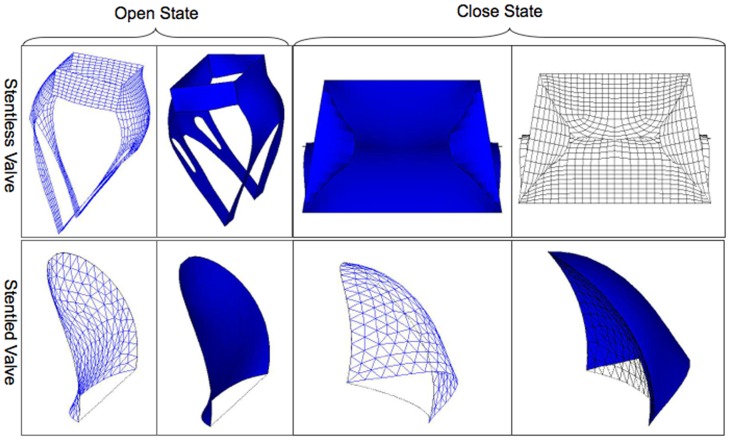
Schematic diagrams of the deformed configuration in the stentless QMV and the stented TMV.

Schematic diagrams of the deformed configuration under open and close conditions are displayed in Figure 3. Valvular deformation relies mostly on the fluid dynamic and the transvalvular pressure gradient. Left ventricular loading starts at the end of left ventricle isovolumic diastole, just after valvular opening, when the finite element model is in the open configuration to achieve blood filling. Left ventricular loading ends at the early stage of left ventricle isovolumic systole, just after valvular closing, when the finite element model is in the close configuration and experiences the maximum transvalvular pressure gradient.

### Stress Analyses

In order to better understand the material stress on the two valve types under multiple conditions, we examined both the moiré patterns and contour diagrams generated by the described models. The pink and red areas of moiré pattern represent high values, whereas blue areas are low values. Similarly, the regions labeled with letter “H” and “I” in contour diagram imply high values, whereas the regions symbolized by “A” are low values.

**Figure 4:**
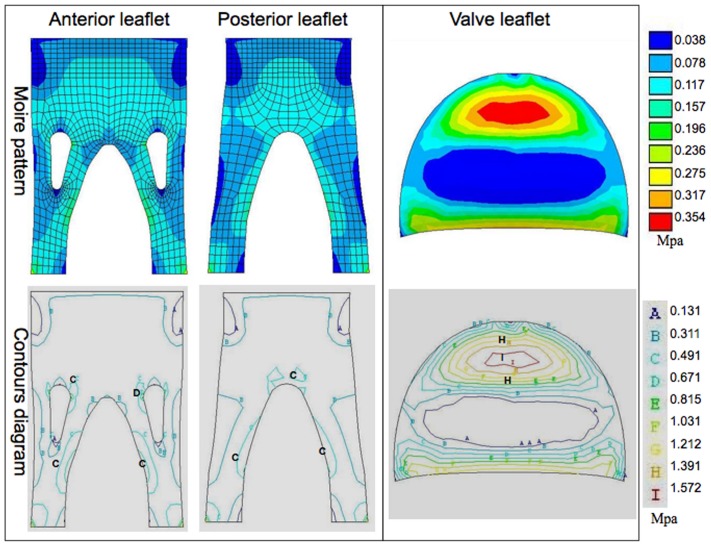
The representative moiré pattern and contour diagram of the first principal stress under the open condition. The red areas represent the high stress, whereas the blue areas are low stress. For the stentless valve (left), limited stress concentration was manifest at the lateral margin of the root of the papillary flaps (regions labeled as “C” and “D”). In the stented valve (right), the region of maximum stress concentration occurred at the central belly (regions labeled as “I” and “H”).

The maximum first principal stresses in the open configuration (Figure 4) for the stentless anterior and posterior leaflets were 0.128±0.046 and 0.132±0.023 Mpa, respectively, while the stented valve showed a maximum first principal stress of 0.201±0.039 Mpa. Consequently, the maximum first principal stress was markedly lower in the stentless valve than in the stented valve in this configuration. Contour diagrams of the stentless valve in the same condition reveal an even stress distribution in the valve leaflets without stress concentration at marked areas; the maximum first principal stress was restricted to the lateral margin of the root of the papillary flaps (regions labeled with “C” and “D”). The region of maximum stress concentration on the leaflets occurred at the central belly in the stented valve (regions labeled with “I” and “H”), and were markedly higher than any region in the stentless valve.

**Figure 5:**
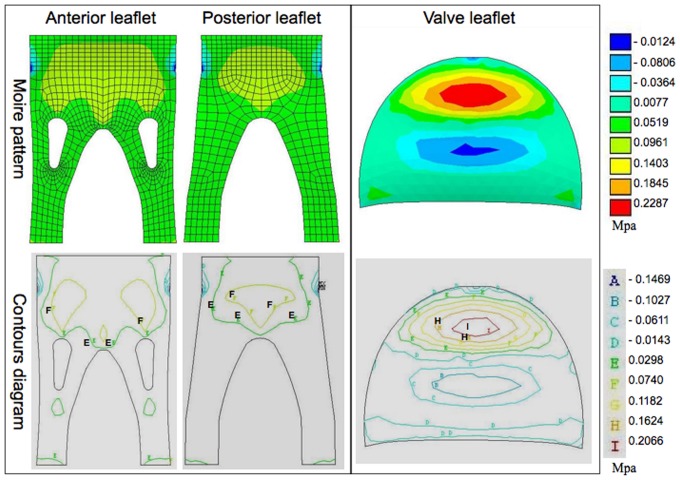
The representative moiré pattern and contour diagram of the second principal stress under the open condition. The red areas represent the high stress, whereas the blue areas are low stress. The stress concentration in the stentless valve (left) is evident at the leaflet belly and free margin by the support of the papillary flaps (regions labeled as “E” and “F”). The maximum second principal stress in the stented valve (right) is located at the center of leaflet belly (regions labeled as “I” and “H”).

The maximum second principal stresses in the open condition for the stentless anterior and posterior leaflets (Figure 5) were 0.088±0.010 and 0.092±0.009 Mpa, respectively. The stented valve showed a maximum second principal stress of 0.215±0.034 Mpa, indicating that, similar to the maximum first principal stress, the maximum second principal stress was significantly lower in the stentless valve than in the stented valve in the open condition. The contour diagram demonstrates that the second principal stress concentration in the stentless valve was evident at the leaflet belly and free margin by the support of the papillary flaps (regions labeled with “E” and “F”). This pattern was similar to that observed in the stented valve, where the maximum second principal stress was observed to be located at the center of the leaflet belly (regions labeled with “I” and “H”).

**Figure 6:**
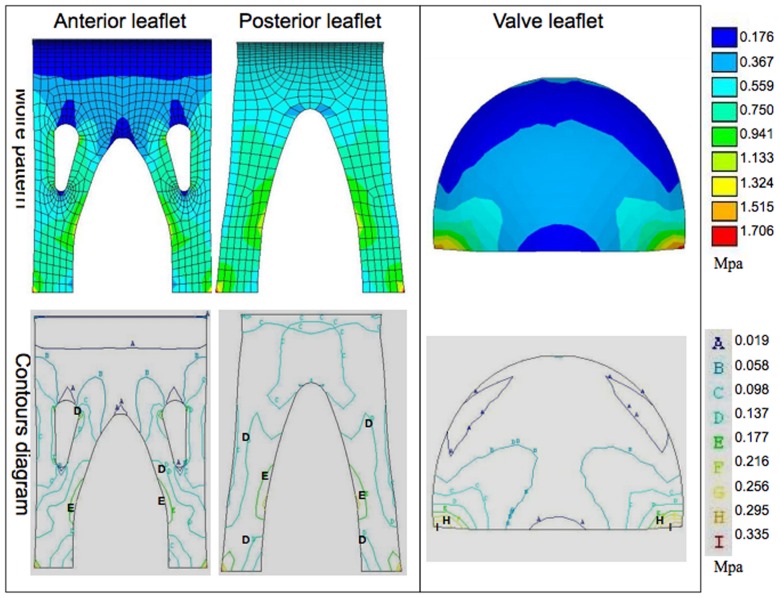
The representative moiré pattern and contour diagram of the first principal stress in the closed condition. The red areas represent the high stress, whereas the blue areas are low stress. Limited stress concentration is discretely distributed in the papillary flaps of the stentless valve (left, regions labeled as “D” and “E”). In contrast, the region of maximum stress concentration for the stented valve (right) is located near the commissural attachment (regions labeled as “I” and “D”).

In the closed configuration, the maximum first principal stresses (Figure 6) for the stentless anterior and posterior leaflets were 0.058±0.009 and 1.341±0.167 Mpa, respectively, compared to 2.19±0.018 Mpa in the stented valve. Thus, the peak first principal stress predicted for the stentless valve in the closed condition represented a very significant reduction compared to that of the stented valve. The contour diagram reveals that stress was evenly distributed across the valve leaflet of the stentless valve in this condition (Figure 6). Nonetheless, limited stress concentration was discretely distributed in the papillary flaps (regions labeled with “D” and “E”). In contrast, the region of maximum stress concentration for the stented valve in the closed condition was observed to be located near the commissural attachment (regions labeled with “I” and “D”).

**Figure 7:**
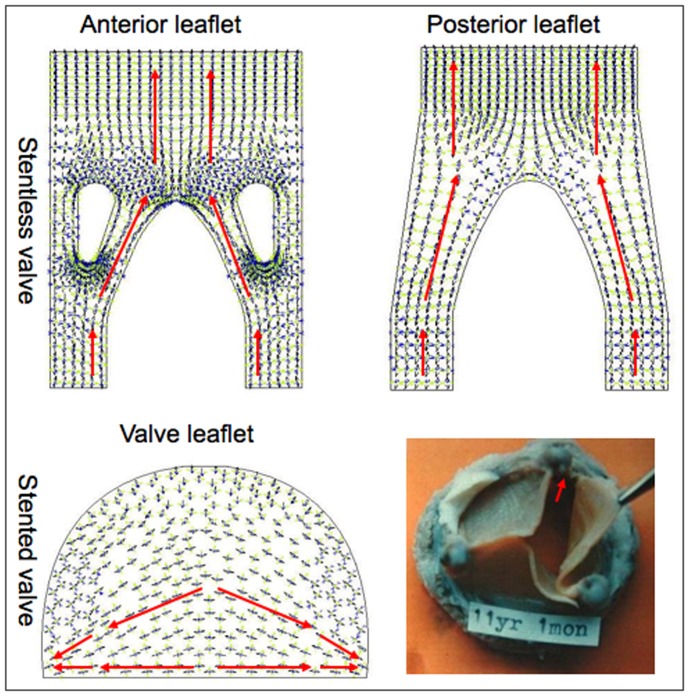
The representative directional diagram of the first principal stress in the closed condition. The red arrowheads indicate the direction of first principal stress conduction within the valve leaflet. Calcification of a stented valve (bottom right) was found most frequently near the commissures (red arrow), less frequently in the belly and free margin, and least frequently at the attachment edge.

We also examined the directional diagram of the first principal stress in the closed condition (Figure 7). The first principal stress reaches its peak value in the closed configuration during the cardiac cycle, which is the leading cause of valvular tearing and perforation. For a stentless valve, the stresses in the longitudinal axis of the valve were primarily transferred from the papillary flaps to the attachment edge. Importantly, the stresses were radically conducted from the central belly to the free margin in the stented valve. More significantly, the directional stresses concentrated on the commissural attachment and were minimally imposed on the attachment edge. The directional distribution of stresses on the stented leaflet coincides with the incidence of calcification of the various regions of the leaflet (Figure 7). Calcification was found most frequently near the commissures, less frequently in the belly and free margin, least frequently at the attachment edge, in complete agreement with the directional stress distribution generated by our model.

**Figure 8:**
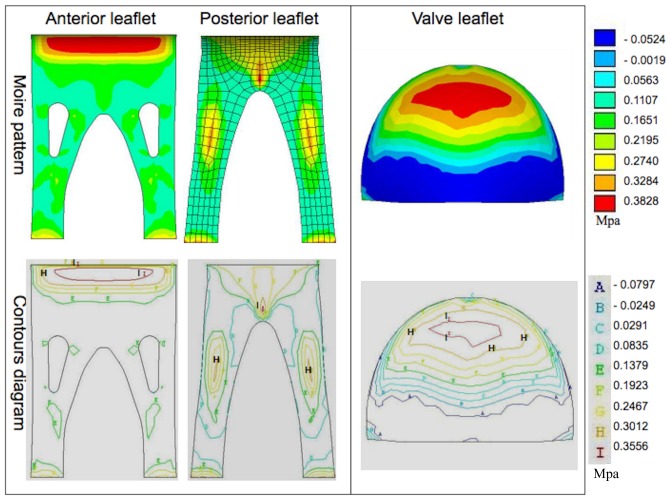
The representative moiré pattern and contour diagram of the second principal stress in the closed condition. The red areas represent the high stress, whereas the blue areas are low stress. For the stentless valve (left), the stress concentration was observed to at the attachment edge in the anterior leaflet and at the middle of papillary flaps in the posterior anterior (regions labeled as “I” and “H”). In the stented valve (right), the region of maximum stress concentration was in the center of the leaflet belly (regions labeled as “I” and “H”).

The maximum second principal stresses in the closed condition (Figure 8) for the stentless valve anterior and posterior leaflets were 0.269±0.01 and 0.417±0.006 Mpa, respectively, and 0.482±0.062 Mpa for the stented valve, indicating a moderately reduced maximum second principal stress in the stentless anterior leaflet compared to the stented valve in this condition. The contour diagram reveals that, for the stentless valve, the stress concentration was observed at the attachment edge in the anterior leaflet and at the middle of papillary flaps in the posterior leaflet (regions labeled with “I” and “H”). The region of maximum second principal stress concentration for the stented valve in the closed condition was observed to be located in the center of the leaflet belly (regions labeled with “I” and “H”).

## Discussion

Presently, no ideal prosthesis for mitral valve replacement is available, and prosthetic valve selection remains controversial. Mechanical valves are associated with increased danger of thromboembolic events, a need for life-long systemic anticoagulation therapy, and an increased risk of intracranial hemorrhages. In contrast, calcification plays a major role in the structural failure of traditional stented biological valves. Increased leaflet stress is one of the contributing factors for bioprosthetic calcification, which adversely affects hemodynamic performance and biological valve durability. Intuitively, replacement with a bioprosthesis resembling native valve anatomy should represent a more durable alternative. Stentless bioprostheses have a flexible annulus, a large opening area, sufficient coaptation, chordal support and a suspension at the papillary muscles to preserve the continuity between the left ventricle and the annulus and to reduce the mechanical stress on the noncoapted leaflets. Thus, they have a sufficiently different geometry from traditional stented valves that we would expect the mechanical stresses on the two valves to be very different.

This finite model study demonstrates that the stentless valve results in a reduction in both tensile and bending stresses in the simulated mitral valve leaflet relative to the stented valve in both the closed and opened configurations. Additionally, the stentless valve displays an even stress distribution and stress concentration mostly occurs in the region of the papillary flaps. Conversely, an overall increase in stress concentration was evident at the belly and commissural attachment in the stented valve. It should be noted that the maximum first principal stress concentrated on the commissural attachment, and the stresses were primarily transferred from the central belly to the commissural attachment in the closed configuration for the stented valve. The directional distribution of stresses on the stented leaflet coincided with the incidence of calcification of the various regions of the leaflet. [3], [4] Calcification was found most frequently near the commissures, less frequently in the belly and free margin, and least frequently at the attachment edge [4].

Our findings were consistent with those observed in a number of previous studies. For instance, Cacciola and colleagues performed a three-dimensional mechanical stress analysis of a stentless aortic valve prosthesis and demonstrated that the stentless aortic valve exhibited a stress reduction of 75% when compared with a stented valve. [14] It has also been previously demonstrated that mechanical stresses in a closed stented valve are highest near the commissural attachments and lowest near the base of the leaflet at a pressure of 100 mmHg, [4] in agreement with the present study.

There is strong evidence to suggest that calcification and failure of bioprosthetics occurs as a consequence of high tensile and bending stresses, which act on the leaflets during opening and closing, [3]–[6] and it has also been suggested that mechanical stress plays a critical role in the rupture of collagen. [15] This rupture is believed to expose the calcium-binding sites of the collagen and initiate the calcification process. [15] Thus, it is of note that our study connects the distribution of mechanical stress on the leaflet directly with the location of calcification of the various regions of the leaflet. [3], [4] Cuspal tears and perforation of bioprotheses frequently occur in the presence of calcification, and they are closely related to impaired long-term performance. [16], [17] Calcification of bioprotheses can be inhibited by reducing leaflet stresses though the optimization of valve design, often to achieve a resemblance to the natural valve anatomy. [5] Considering the documented decrease in leaflet stress resulting from the absence of a rigid stent, we postulate that a stentless valve might have a lower incidence of tearing and perforation, and may demonstrate an improved durability in the clinic.

A variety of clinical studies support the beneficial value of a stentless mitral valeve. For instance, a stentless quadrileaflet mitral valve (Quattro™; St. Jude Medical Inc., St. Paul, MN, USA) has been clinically implanted in several cardiac centers and has presented good clinical outcomes and excellent hemodynamic performance. Vrandecic and colleagues performed follow-up studies at a mean of 28.5 months in 120 patients undergoing stentless valve replacement, and their studies indicate that this technique was beneficial for preserving annuloventricular continuity and improving left ventricular function. [18] In addition, a single center prospective study involving 38 patients demonstrated that stentless QMV replacement increased cardiac output, lowered transmitral pressure gradients, and significantly improved left ventricle function. [19] Moreover, Walther and colleagues investigated 20 patients one year after implantation of the stentless QMV and found that all patients displayed as NYHA functional class I or II and had normal postoperative mitral valve function. [20] Another study reported the preliminary experience of 22 patients undergoing the stentless QMV replacement and showed that the postoperative cardiac index was improved significantly and that hemodynamic performance of the novel bioprosthesis was favorable. [21] Aybek and colleagues reported a progressive improvement to NYHA class I or II and a consistent decrease in transvalvular pressure gradients at 3–6 months and 12 months postoperatively in 15 patients, [22] while Hofmann and colleagues reported that intraoperative and postoperative echocardiography showed an excellent valve function of the stentless QMV in 12 patients. [23] Finally, it was reported that restitution of physiological cardiac function was achieved by combined stentless QMV implantation and left atrial ablation therapy. [24] Therefore, the stentless QMV replacement is clinically feasible and practical, and offers an advantageous alternative to conventional biological mitral valve implantation.

As with any modeling study, there are some inherent limitations in our model. First, because of software restrictions, the material properties were believed to be constant in the physiologic range of the stress-strain curve. Consequently, the modeled stresses and strains were likely lower than those in the actual valve. Second, the analysis believed that the initial zero-pressure state in the valve was identical to a zero-stress state. Because of normal dynamic valve motion, a zero-stress state actually does not exist in vivo. Third, a deformation analysis was performed based on the rationale that the applied simulated pressures did not result in significant changes in the model geometry. We therefore postulated that any incrementally deformations would not be large enough to justify extracomputational burden.

In conclusion, the stentless QMV was associated with a significant reduction in leaflet stress and a more homogeneous stress distribution as compared with the traditional stented TMV. These findings are consistent with the many reports of positive clinical outcomes using stentless QMVs, and indicate that the reduced stress of this valve type makes it superior to traditional stented valves for clinical use.
